# Randomized controlled trial of computer-based treatment of social cognition in schizophrenia: the TRuSST trial protocol

**DOI:** 10.1186/s12888-015-0510-1

**Published:** 2015-07-03

**Authors:** Annika Rose, Sophia Vinogradov, Melissa Fisher, Michael F. Green, Joseph Ventura, Christine Hooker, Michael Merzenich, Mor Nahum

**Affiliations:** 1 Posit Science Corporation, 77 Geary Street, San Francisco, CA 94108 USA; 2 San Francisco Veterans Affairs Medical Center, 4150 Clement Street, San Francisco, CA 94143 USA; 3 VA Greater Los Angeles, 11301 Wilshire Boulevard, Los Angeles, CA 90073 USA; 4 UCLA Aftercare Research Program, 760 Westwood Plaza, Los Angeles, CA 90095 USA; 5 Department of Psychology, Harvard University, 1020 William James Hall 33 Kirkland St., Cambridge, MA 02138 USA

**Keywords:** Schizophrenia, Rehabilitation, Social cognition, Computer-based cognitive training, Clinical trial

## Abstract

**Background:**

Schizophrenia is a severe and chronic medical condition, characterized by positive and negative symptoms, as well as pervasive social cognitive deficits. Despite the functional significance of the social cognition deficits affecting many aspects of daily living, such as social relationships, occupational status, and independent living, there is still no effective treatment option for these deficits, which is applied as standard of care. To address this need, we developed a novel, internet-based training program that targets social cognition deficits in schizophrenia (SocialVille). Preliminary studies demonstrate the feasibility and initial efficacy of Socialville in schizophrenia patients (Nahum et al., 2014). The purpose of the current trial (referred to as the TReatment of Social cognition in Schizophrenia Trial or *TRuSST*) is to compare SocialVille to an active control training condition, include a larger sample of patients, and assess both social cognitive functioning, and functional outcomes.

**Methods/Design:**

We will employ a multi-site, longitudinal, blinded, randomized controlled trial (RCT) design with a target sample of 128 patients with schizophrenia. Patients will perform, at their home or in clinic, 40 sessions of either the SocialVille training program or an active control computer game condition. Each session will last for 40–45 minutes/day, performed 3–5 days a week, over 10–12 weeks, totaling to 30 hours of training. Patients will be assessed on a battery of social cognitive, social functioning and functional outcomes immediately before training, mid-way through training (after 20 training sessions) and at the completion of the 40 training sessions.

**Discussion:**

The strengths of this protocol are that it tests an innovative, internet-based treatment that targets fundamental social cognitive deficits in schizophrenia, employs a highly sensitive and extensive battery of functional outcome measures, and incorporates a large sample size in an RCT design.

**Trial Registration:**

ClinicalTrials.gov NCT02246426Registered 16 September 2014

## Background

Schizophrenia is a severe, chronic mental illness that affects more than two million individuals in the U.S. [1]. Individuals with schizophrenia have both positive and negative symptoms, including hallucinations, delusions, disorganized speech and behavior, alogia (poverty of speech), affect flattening, and avolition (inability to initiate and persist in goal-directed behaviors) [2]. These clinical symptoms are often accompanied by severe cognitive deficits, in speed of processing, attention, working memory, verbal and visual learning and memory, and executive function [3]. Moreover, individuals with schizophrenia often exhibit difficulties in social functioning, namely in their ability to navigate through the social world, create meaningful interactions, and correctly interpret relevant social context [4]. This poor social functioning has been attributable to pervasive and enduring impairments in*social cognition* [5–10]: the perception, interpretation and processing of socially-relevant information [11–16]. Individuals with schizophrenia exhibit deficits in all core domains of social cognition [17, 18]: *emotion perception* (the recognition of facial and vocal affect) [19–23], *social cue perception* (the ability to detect and comprehend cues in a social context) [24–26], theory of mind (the mental capacity to infer one’s own and others’ mental states) [1, 27–29], *attributional style* (attribution of causes of events to the self, to others, or to factors in the environment) [30, 31], and *empathy* (the ability to share, understand and appropriately react to the emotional states of others) [32]. Recent studies have shown that these social cognitive deficits in schizophrenia are rooted in anatomical and functional abnormalities within a complex brain network collectively termed “the social brain” [33–39], and include the superior temporal sulcus (STS), anterior insula, amygdala, medial prefrontal cortex (mPFC), and to the cingulate cortex [40–44]. These fundamental, multi-domain social cognition impairments are not only directly linked with poor *social* functioning, but also underlie most critical factors of *daily living* in schizophrenia, such as low occupational status, poor social and community functioning, reduced capabilities for independent living, high relapse rate, and reduced quality of life [2, 4, 45–51]. Moreover, the degree of social cognition impairment is a stronger predictor of the level of everyday functional ability than are cognitive abilities or the severity of positive symptoms [52, 53]. This makes social cognition an important treatment target in schizophrenia: as the ultimate goal of therapeutic interventions is to improve life outcomes for patients, it is now clear that recovery of these individuals to the broader society is crucially dependent upon the recovery of their social cognitive abilities. The fact that social cognition deficits persist throughout the course of the illness [22, 54, 55], are seen in prodromal patients [22], and are even present in unaffected relatives of patients, further stresses their central role in schizophrenia and fuels the need for an effective, scalable treatment for social cognitive deficits (see recent review in [44]).

Despite the importance of social cognition as a primary source of impairment, there are currently no well-accepted or even broadly administered treatment methods for improving social cognitive function in schizophrenia patients. Social cognitive deficits are resistant to pharmacological treatments including second-generation antipsychotic medications [56–60] that are effective for controlling positive symptom levels [58]. Perhaps more surprisingly, new and demonstrably effective interventions for treating cognitive deficits in schizophrenia have been shown to have only limited impacts on social functioning [61] - presumably because social cognition deficits are associated with impaired function of neural networks that are largely distinct from, and parallel to, those subserving general neurocognition [18].

Several experimental, therapist-delivered approaches targeting social skills or social cognition have been developed over the last decade (e.g. [62–66]), and initial studies have shown some promising results (see recent reviews in [52, 67, 68]). These interventions are offered in only a few clinics nationwide and are usually administered by trained professionals individually or in small groups over the course of several months. The therapist-administered options (e.g. Social Cognition and Interaction Training (SCIT) [66, 69–71]; Social Cognitive Skills Training (SCST) [72]; Emotion and ToM Imitation Training (ETIT) [73]; Social Cognition Enhancement Training (SCET) [74]) usually focus on emotion management and social skills building, and require multiple in-person visits to the clinic in the course of a few months. Recently, several computer-aided interventions (e.g. Tackling Affect Recognition (TAR) [75]; MicroExpression Training Tool (METT) [65]) have been created. These interventions are limited in scope (mainly target a single social cognitive domain in isolation, such as facial affect recognition), have undergone only initial testing in schizophrenia [52, 65, 75], and are not used, to the best of our knowledge, in any clinical treatment programs. While, collectively, these approaches show promise for social cognitive treatment in schizophrenia, to date, no single treatment has been widely adopted, and there is no standard of care for social cognitive treatment in schizophrenia. This is potentially due to the fact that these interventions are not scalable and cost-effective, as they require highly-trained personnel and necessitate frequent visits to the clinic, limiting their scalability and significantly increasing their associated costs.

To address the need for a scalable and effective treatment for social cognition deficits in schizophrenia, that considers these deficits from their neurological core (see [44, 76, 77] for recent reviews on this topic), we have developed SocialVille, an internet-based treatment program designed to specifically address the core social cognitive domains of deficit in individuals with schizophrenia. While the use of computer-based strategies to strengthen social behaviors may seem paradoxical or counter-intuitive, the goal of this form of training does not entail strengthening explicit social *skills*, but rather strengthening the brain basis that comprises these skills, for which the use of a computer is a substantial advantage. The 27 different exercises of SocialVille collectively encompass the five social cognitive domains (see Table 1 for a complete list). The user is required to make hundreds of speeded, accurate, and increasingly more challenging discriminations of socially-relevant information (e.g., emotional faces, eye gazes, prosody, social situations). During training, the user is systematically exposed to socially-relevant stimuli, starting from very basic-level stimuli and gradually involving more complex, multi-modal, and ecologically-valid stimuli. Trial-by-trial difficulty is adaptively set using either up-down [78] or Bayesian [79] algorithms, maintaining individual success rate at 70-80 % success level, allowing for progression through training based on the user’s individual performance level. Finally, a secure online clinician portal allows the treating clinician to track user performance and treatment compliance.

**Table 1:** SocialVille training exercise

Exercise	Description
*Affect Perception*	
Name That Feeling	Select the label which correctly describes the target facial affect (stills)
Face It	Identify the target face within a group of faces
Match that Feeling	Match the facial affect of the target face with that of an array of different faces
Voice Choice	Select the label which correctly describes the target vocal affect (prosody)
Second That Emotion	Match pairs of cards that express the same facial affect
Second That Intonation	Match pairs of cards that express the same vocal affect (prosody)
Emotion Motion	Select the label which correctly describes the target facial affect (video clips)
Emotion Motion:Flashback	Memorize a sequence of facial expressions (video clips)
*Social Cue Perception*	
Recognition	Select the target face from an array of neural faces
Face It: Flashback	Memorize a sequence of faces
Gaze Cast	Follow the gaze shift of a person, to track the peripheral object looked at
Gaze Match	Select the target gaze from an array of gazes (irrespective of face identity)
Life Stories	Answer questions regarding social cues after listening to a segmented story
Face Facts	Memorize visually-presented social facts about individuals presented serially
In the Know	Memorize aurally-presented social facts about individuals
Face and Name	Memorize pairs of faces and names
Pragmatic Ambiguity	Determine if the given conversation makes sense (visual scene)
Social Skills	Choose the best way to respond during a difficult conversation
*Self-Referential Style*	
Mass Affect	Memorize internally-generated valence labels over time
Bright Whites	Identify which of two people displayed the more positive affect
Grin Hunting	Select the more positive scene to detect smiles in a subsequent image
*Theory of Mind*	
Social Scenes	Rate the likehood of people’s reactions and feelings in social situations
What Joe’s Thinking?	Track a person’s gaze shift to determine if they are looking at the same object as the person in their line of view
What Happened?	Determine the most likely scenario given the least amount of hints
Say What?	Decide how would a person would respond in a given situation (audio scene)
Person Description	Infer what someone believes based on given facts about them.
*Empathy*	
Multi-person Perspective-Taking	Decide how a person will be affected by a given situation (visual scene)

Two recently-published studies using SocialVille show promising initial results in both adults with schizophrenia [80] and in young adults at high clinical risk for psychosis [81]. Nahum et al. found that following 24 hours of SocialVille training, participants showed improvements in proximal measures of social cognition (e.g. facial memory, prosody identification), as well as in more remote measures of social functioning and motivation [80]. These preliminary results of SocialVille demonstrate some transfer of training benefits to more general skills. The current multi-site clinical trial extends this and other studies with a longer duration of SocialVille training (30 hours total), the inclusion of an active control training condition (computer games, see Table 2), and the inclusion of additional social and functional outcome measures.

**Table 2:** Active cotrol training games

Game	Description
Chinese Checkers	Move your pieces to the opponent’s end by moving or jumping over pieces
Sudoko	Fill each square in the puzzle with a number (1-9) given rules
Reversi	Try to have the majority of the disks on the game present your color
Double Klondike Solitaire	Participants must stacks cards alternating in color in descending order with the goal of forming complete A-K stacks of the same suit.
Tri Peaks Solitaire	The object of the game is to remove all cards that make up the “three peaks.” Player must stack the cards present on the ‘peaks’ to the card on the bottom
Brick Breaking Hex	Click on a group of blocks with the same color. To remove individual blocks, you lose one of your stars. The goal is to get rid of all the blocks before you lose all your stars
Brick Squasher II	Use the mouse to control the board to bounce the balls and destroy the bricks. Some bricks require a few hits and some bricks are indestructible
Gem Swap	Swap adjacent gems to create 3 or more in a row to remove the gems
War Ship	Hide your ships then take turns with the computer player to search for the opponent’s hidden ship. The object of the game is to find your opponent’s ships and sink them before they find yours
A Maze Race	There are two balls, the green one is designated to the participant and the red is the computer player. The participant must find the ‘Flag’ or end point before the computer does
Lineup 4	Participant and computer player take turns dropping discs from the top into a grid. The player must connect four yellow discs in a row (vertically, horizontally or diagonally) before the opponent
Word Search	Letters are placed in a grid and the participant must find the specified list of words hidden within the grid

Two additional innovations in the current protocol are worth mentioning. First, to the best of our knowledge, this trial is the first to include a fully-Internet-based treatment for social cognitive deficits in schizophrenia, designed to be completed entirely remotely (from home), with minimal supervision and clinic visits only for assessments (see recent review of training studies in [82]). Some studies employing social cognition training in schizophrenia have used computerized interventions, but these were usually applied as part of a larger, instruction-based therapy [66, 69–72, 83] or in small groups of participants in the clinic (e.g. [84]). The advantage of using a fully online training program is that it can be adaptively tailored to patient’s individual abilities to provide the appropriate level of training, and is easily scalable to support many users.

The second innovation in our current protocol is the use of a large battery of outcome measures, including novel, computerized measures (e.g. Virtual Reality Functional Capacity Assessment Tool (VRFCAT) [85]). As the ultimate goal of clinical intervention is to improve functional outcomes by improving social abilities, it is important to document the degree of improvement in various aspects, encompassing social and global functioning, quality of life, functional capacity, motivation, and symptom severity level. Nonetheless, previous studies generally employed limited batteries of outcome measures (see [67, 68]) or lacked adequate controls that are matched for intensity and experimenter contact [86].

### Aims and hypotheses

The aim of the current study is to test the effectiveness of 30 hours of computer-based SocialVille training to improve social cognition and functional outcome in schizophrenia, compared with an active control intervention. Based on our previous findings, we predict that the SocialVille group will show statistically greater gains on co-primary outcome measures of social cognitive performance and functional performance as well as secondary measures of social cognition, functional capacity, functional outcome, motivation, and quality of life, indicating that SocialVille drives generalized social cognitive improvements as well as real-world functional improvements. Secondary aims include determining which patients best respond to SocialVille, and evaluating the effects of SocialVille training on low-level vs. high-level social cognition factors. For treatment response, we will examine predictors of social cognitive gains based on baseline participant demographic, symptom severity level, prior computer use, and functional measures, as well as on learning rate and plateau performance measures derived over the course of SocialVille use. Based on our previous studies, we hypothesize that most baseline measures will not define responder/non-responder groups effectively (except potentially for symptom severity), while baseline exercise performance, learning rate and plateau performance during SocialVille use will predict overall gains. Finally, we will separately examine the effects of training on the independent social cognitive factors of low-level social cue detection and high-level inferential process. We hypothesize that both low-level and high-level social cognition factors will be significantly correlated with functional capacity and real-world social and role functioning.

## Methods

### Ethics Statement

The Western International Review Board (WIRB) is designated to review and provide continuing oversight of ethical standards involving human subjects research (WIRB Pro Number 20141695). Research is conducted in accordance with the Declaration of Helsinki and monitored by the WIRB. Participants interested in the study will meet with qualified study staff for the consenting process, during which the participant is informed of the nature of the trial, purpose of research, trial procedures, risks and benefits, confidentiality, etc. Following consent, the participant will be assessed for eligibility and potential enrollment in the trial. Minors are excluded from this study and will not undergo the consenting process.

### Overall Design and Timeline

The current study will employ a multi-site, longitudinal, blinded randomized controlled trial (RCT) design with a target sample of 128 patients with schizophrenia (see inclusion criteria below). This trial will follow the Consolidated Standards of Reporting Trials (CONSORT) guidelines [87] for the design, execution, and reporting of clinical trials, with the Non-Pharmacologic Treatment Interventions extensions to reflect the use of computerized social cognitive remediation as a treatment intervention.

**Fig. 1:**
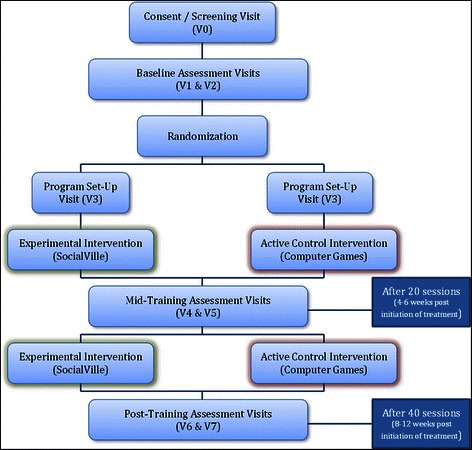
Study Outline. Following screening and baseline visits, participants are randomized into the experimental intervention (SocialVille) or the active control intervention (AC), in which they complete 40 sessions of training, with assessment visits conducted after 20 sessions and after training completion

Sixty-four patients with schizophrenia completing SocialVille will be compared to sixty-four patients with schizophrenia completing the active control condition (online computer-based games; see Fig. 1). Total participation time is approximately 12–14 weeks and includes seven (7) in-person assessment sessions. The first assessment session (V0) involves screening for eligibility (see inclusion/exclusion below). If the participant is eligible, they next perform two baseline assessment visit (V1-V2) to provide baseline level of their symptoms, social cognition, social functioning, functional capacity and motivation before training. After the baseline assessments, patients are randomized to either the SocialVille or control training program, and complete a set-up visit (V3) in which they are provided with a study laptop with their corresponding training program. Participants are then requested to complete 40 training sessions (about 10–12 weeks) of in-home training, while monitored remotely by a research assistant (cognitive remediation coach). Participants will be assessed again at the completion of 20 sessions (mid-intervention assessments; V4-V5) and at the completion of the entire training of 40 sessions (post-intervention assessments, V6-V7), to measure potential training-related improvements. After this visit (V7), participant activities are completed and trial participation ends. All assessments will be done by assessors that are blind to group affiliation.

### Study Population

The study population is comprised of individuals diagnosed with schizophrenia. All participants must be clinically stable and be stable on the doses of the psychiatric medications they are taking. For the purposes of this trial, we will focus on treating core symptoms rather than examining etiology, and only enroll those individuals that meet inclusion/exclusion criteria (see below).

We will employ a multi-site study, which is used to ensure that the results are not particular to the participant population or study operations at a single site. We have ongoing research collaborations with all site PIs (who served as consultants and collaborators on the Phase I grant). Study participants will be recruited with established and proven mechanisms developed at each site. All Site PIs, internationally-recognized experts in the field, have extensive expertise in this population as well as access to large cohorts of patients with schizophrenia.

We do not anticipate any specific barriers to the accrual of participants, nor are we aware of a large number of competing clinical trials that would limit enrollment. The study population is limited to those 18 years of age or older. We chose a minimum age of 18, to match the minimum age for participation without parental consent, and since schizophrenia is usually seen in adults above this age. The study is open to all races, ethnicities, and genders. Selection of participants is based on psychiatric condition and is not based on gender or ethnic considerations, although these are expected to reflect the diverse population of San Francisco Bay Area, Los Angeles, and Boston. Ethnic minorities will be included when available and recruiting efforts will target a balanced enrollment.

The following inclusion/exclusion criteria will be determined through our screening procedures during V0, which includes structured interviews, as well as computerized and standardized neuropsychological assessments of attention, cognition and functional abilities.

#### Inclusion Criteria

1. Subjects must be between 18 and 65 years old at the time of study screening
2. Subjects must have a diagnosis of schizophrenia as defined by DSM-V criteria and confirmed by the Structured Clinical Interview for DSM-IV (SCID-P [88]). The SCID-P is a semi-structured clinical interview used to determine major mental disorders and personality disorders and will be used to confirm diagnosis of schizophrenia.
3. Subjects must demonstrate adequate decisional capacity, in the judgment of the consenting study staff member, to make a choice about participating in this research study.
4. Subjects must have been clinically stable (non-acute) for 8 weeks prior to consent; in the judgment of the Site Principal Investigator.
5. Subjects must have been maintained on a stable treatment of antipsychotics and/or other concomitant psychotropic treatment for at least 6 weeks prior to consent.
6. Subjects must have learned English before the age of 12 to ensure valid neuropsychological results.
7. Subjects must have the visual, auditory, and motor capacity to use the computerized intervention in the judgment of the consenting study staff person.
8. Subjects must have no more than a moderate severity rating on hallucinations and unusual thought content as shown by a score of  ≤ 4 on the Positive and Negative Symptoms Scale (PANSS [89]).

#### Exclusion criteria

1. Subjects should not have had a psychiatric hospitalization in the 8 weeks prior to consent.
2. Subjects who appear to be intoxicated or under the influence of a controlled substance on any day of assessment must be rescheduled or discontinued based upon the discretion of the site staff evaluator.
3. Subjects should not have a history of mental retardation (IQ < 70 based on The Wechsler Test of Adult Reading, WTAR [90]) or pervasive developmental disorder; or other neurological disorder (e.g., Traumatic Brain Injury, epilepsy, Parkinson’s Disease)
4. Subjects should not have been treated within 5 years of the date of consent with a computer-based cognitive training program manufactured by Posit Science.
5. Subjects should not be participating in a concurrent clinical trial that, in the judgment of the Site Principal Investigator, could affect the outcome of this one.
6. Subjects should not be prescribed more than two anti-psychotics. Subjects should not be treated with medication(s) with a total Cogentin equivalent greater than 4.5 mg (known anti-cholinergic side effects)
7. Subjects who have answered ‘yes’ to Question 5 (Active Suicidal Ideation with Specific Plan and Intent) on the Columbia-Suicide Severity Rating Scale (C-SSRS [91]), or who have answered ‘yes’ to any of the suicide-related behaviors (actual attempt, interrupted attempt, aborted attempt, preparatory act or behavior) on the C-SSRS “Suicidal Behavior” portion shall be excluded from the study if ideation or behavior occurred within two months of consent. Subjects excluded for this reason will be referred for appropriate treatment.

### Repeated Assessment Battery (Outcome Measures)

Once a participant is deemed eligible for participation based on their V0 results, they are next scheduled for their baseline sessions on the repeated assessment battery (V1-V2), which take approximately four hours total. After completing these baseline assessments, participants are randomly assigned to either experimental or control training conditions (see below). Then, after half of the training is completed (20 sessions), participants are given the repeated assessment battery (V4-V5), and again at the completion of the entire training protocol (20 additional sessions for a total of 40 sessions; V6-V7).

We will employ a battery of neuropsychological and functional assessments (see Table 3), measuring a range of parameters from proximal to very distal from program use (e.g., assessment of functional abilities and quality of life). This structure will allow us to determine the degree of transfer of benefit to untrained modalities, including the extent to which improvements generalize to untrained functional ability and real-world experience (e.g., quality of life). Assessments will be administered to all participants, including those enrolled in treatment and active control groups; alternate forms of the assessments will be used when available to mitigate test-retest effects. Site psychometricians conducting the assessments will be blinded to group allocation and will receive training by a member of the coordinating center staff regarding appropriate administration of all measures, scoring and data reporting to ensure data quality. Performance on all measures will be scored, submitted by the participating site into the study database, and monitored for accuracy and integrity by the Coordinating Center.

**Table 3:** TRuSST Study primary and secondary outcome measures

Domain	Outcome measure
*Co-Primary Outcome Measures*	
Social Cognition	Penn Emotion Recognition Test (ER40)
	Prosody Identification (PROID)
	Penn Facial Memory Test (PFMT)
	MSCEIT-managing emotions subscale
	The Empathic Accuracy (EA)
Functional Capacity	The UCSD Performance-based Skills Assessment (UPSA-2)
*Secondary Outcome Measures*	
Symptom Severity	Positive and Negative Syndrome Scale (PANSS)
Functioning	Global Functioning Scale
	Social Functioning Scale
	The Specific Levels of Function (SLOF)
	VRFCAT
Social Cognition	The Awareness of Social Inference Test (TASIT), Part 3
	The Morphed Faces Task The Faux Pas Test
	The Source Memory Test
	The Ambiguous Intentions Hostility Questionnaire (AIHQ)
Motivation	Behavioral Inhibition/Behavioral Activation Scale (BIS-BAS)
	Temporal Experience of Pleasure Scale (TEPS)
Quality of Life	The Quality of Life Scale (QLS)

### Primary Outcome Measures

The FDA has indicated through the MATRICS (Measurement and Treatment Research to Improve Cognition in Schizophrenia) guidelines that it requires two co-primary endpoints for trials of cognitive enhancement in schizophrenia, a cognitive endpoint and a functional endpoint. We will follow these recommendations and include two co-primary outcome measures, a social cognitive outcome measure and a functional capacity measure. Our selections also adhere to the NIH toolbox recommendations regarding assessments, as this trial is sponsored by the National Institute of Mental Health (NIMH).

#### Social Cognitive Outcome Measure

The a priori co-primary social cognitive outcome measure will be a composite score comprised of a standardized, validated set of assessments that tap into low-level and high-level SC (social cognition) abilities (see [92]). A composite score will be derived from all five assessments to provide a social cognitive co-primary measure which encompasses several SC abilities. All assessments have been validated and used in outcome studies [68].

The low-level social cognitive assessments are:

1. ER40 (The Penn Emotional Recognition Test)[93] is a computerized test to assess categorical identification of facial emotions. The tasks consists of 40 digital pictures of faces. The photos are presented individually and subjects are required to choose the most appropriate emotion label from five possible emotion choices (happiness, sadness, anger, fear, or no emotion). The 40 photos are of 8 actors, and there are 8 photos of neutral expressions, 4 low intensity and 4 high intensity photos for each emotion. The index of accuracy is the total number of correct items and response time. This measure was found to exhibit 70% accuracy of identifying emotions, with concurrent validity of .83, convergent validity of .79 and divergent validity of .09.
2. PROID (Prosody Identification)[94] is a computerized vocal identification task used to assess a subject’s ability to perceive and discriminate emotion in the speech of others. All stimuli have been validated through trials with healthy controls.
3. PFMT (Penn Faces Memory Test)[93] consists of 20 target faces and 40 foil faces; Stimuli are black and white photographs of faces, balanced for gender and age. All faces are of neutral expression. Participants are requested to view the pictures, and are then tested immediately and after a delay on the pictures they have seen, to determine if they were in the set they memorized or not.

The high-level social cognitive measures are:

1. MSCEIT (Mayer-Salovey-Caruso Emotional Intelligence Test)[95] managing emotions subscale has two subtests that assess how participants manage the emotions of others (Social Management) and how a person would regulate his or her own emotions (Emotion Management). The test-retest reliability has been found to be .86 over a three-week interval and the full-test split-half reliabilities range from .91-.93. Discriminant and convergent validity has been demonstrated.
2. The EA Task (Empathic Accuracy) [32] is an empathy measure, in which subjects are shown multimodal video stimuli and are required to make continuous inferences about a target’s specific thoughts and feelings, which are later compared to the targets’ reported actual thoughts and feelings in order to compute an index of the perceiver’s accuracy. Empathic accuracy has been shown to demonstrate adequate test-retest reliability (.72).

#### Functional Capacity Outcome Measure

The a priori co-primary *functional* measure will be the functional capacity measure of The UCSD Performance-based Skills Assessment (UPSA-2)[96].The UPSA is a well-validated measure frequently used in cognitive and SC training studies [68]; In addition, it measures functional capacity, which is expected to be affected by social cognitive change. The UPSA-2 is designed to assess skills in five areas (Household Chores, Communication, Finance, Transportation, and Planning Recreational Activities) that reflect general abilities that are important components of independent living. Test-retest reliability ranged from .63-.80 over follow-up periods up to 36 months in patients with schizophrenia. Among patients, the UPSA performance correlated significantly with severity of negative symptoms and of cognitive impairment but not with that of positive or depressive symptoms.

### Secondary Outcome Measures

The following validated and normed assessments will be used as secondary outcome measures in the study:

#### Clinical Status and Symptom Severity

We will use the PANSS (Positive and Negative Syndrome Scale) [89] to assess clinical status and severity of symptoms. The PANSS is a clinically-administered exam used for measuring symptom severity of patients with schizophrenia of 30 different symptoms, divided into Positive, Negative, and General Psychopathology scales. These scales have been found by coefficient alpha, split-half method, and test-retest reliability testing to be internally consistent and highly reliable [97].

#### Functioning

The following set of outcome measures will be used to assess global and social functioning:

1. GFS (Global Functioning Scale)[98, 99] is a clinician-administered questionnaire which is used to assess general psychosocial functioning. It has two subscales: role and social.
2. SFS (Social Functioning Scale)[100] is a self-report questionnaire designed to assess social functioning in individuals with schizophrenia. To be completed by both the subject and a relative, the scale is made up of 54 questions divided into seven sections. Results from three samples show the SFS is reliable, valid, sensitive and responsive to change.
3. SLOF (Specific Levels of Functioning)[101] is a 43-item scale designed to assess in detail an individual’s basic living skills and level of independent functioning. Item reliabilities are .62 in community programs and 0.42 in state hospital; internal consistency > =.91.
4. VRFCAT (Virtual Reality Functional Capacity Assessment)[85] is a virtual reality measure mimicking a real-life scenario of a shopping trip. The test has several alternate forms and it records number of errors and RT for task completion.
5. The QLS (Quality of Life Scale)[102] will be used as a secondary outcome measure to assess quality of life. QLS is a 16-item instrument used to measure five conceptual domains of quality of life. It has low to moderate correlations with physical health status and disease measures; however, content validity analysis indicates that the instrument measures domains that diverse patient groups with chronic illness define as quality of life.

#### Motivation

The following secondary outcome measures will be used to assess motivation:

1. BIS/BAS (Behavioral Inhibition/Behavioral Activation Scale)[103] is a 24-item self-report questionnaire designed to assess the two general motivational systems that underlie behavior and affect, i.e. sensitivity to anticipated punishment or reward. Test-retest correlations were found to range from .59-.69.
2. TEPS (Temporal Experience of Pleasure Scale)[104] is a measure specifically designed to capture the anticipatory and consummatory facets of pleasure. The 10-item anticipatory pleasure scale and 8-item consummatory pleasure scale were found to be internally consistent, temporally stable and moderately, positively correlated with each other.

#### Social Cognition

The following social cognition measures will be used as secondary outcome measures, in addition to the primary social cognitive outcome measures:

1. TASIT (The Awareness of Social Interaction Test)[105], part 3. The TASIT is a social perception test in which subjects watch scenes which involve lie/sarcasm and are asked questions about them; We will use the 3^rd^ part of the test, the Social Interference Enriched test. Test-retest reliability has been found to range from .74-.88 and alternative forms reliability ranges from .62-.83.
2. The Morphed Faces task [106] is a computerized emotion perception task, in which participants are presented with faces that are morphed between a neutral expression and an emotional expression: happy, disgusted, angry, or fearful. All faces are morphed between a neutral expression and either 20, 30, 40, 50, or 60 of the emotional expression, and are created from 1 male, and 2 female targets, resulting in a total of 60 face stimuli (3 targets, 5 levels of morph, 4 emotions).
3. The Faux Pas test [107] will be used as a Theory of Mind (ToM) measure. The test is comprised of 20 short stories, incidents of faux pas (someone mistakenly saying something they shouldn't have). Stories are read to the individual, who is then asked questions to determine whether or not they recognized the faux pas.
4. The Source Memory Test [31] is a measure of memory for the source of self-generated, and experimenter-provided word items that shows strong associations to social cognition[108]
5. AIHQ (The Ambiguous Intentions Hostility Questionnaire)[109] is a measure of attributional style, and specifically of hostile social-cognitive biases comprised of a variety of negative situations that differ in terms of intentionality. This measure has demonstrated good levels of internal consistency and inter-rater reliability, and is positively correlated with paranoia and hostility but not correlated with measures of psychosis proneness (convergent and discriminant validity).

### Randomization

Participants will be randomized after the last baseline visit (V2) and before the planned program set-up visit (V3), which is the first day of program use. All V0-V2 data for each participant must be fully monitored, with all queries resolved, before randomization may take place.

Given the potential importance of cognitive abilities on the response to social cognitive training, participants will be stratified by gender, education (<13 years, >13 years) and age (18–40 years, 41–65 years) and randomly assigned to either treatment (SocialVille) or active control group at each site with an allocation ratio of 1:1. We will employ a minimization method of adaptive stratified randomization (referred to as the ‘platinum standard’ of randomization methods when stratification is required) to minimize the imbalance between the number of participants in each group over these factors.

For this trial, we will use a secure randomization server (Sealed Envelope) that implements the specified procedure and an unblinded study member Site Coordinating Center will issue a randomization assignment at the appropriate time. This approach represents a best practice approach to randomization, implementing an automated centralized group assignment procedure with allocation concealment, and effective separation of sequence generation and allocation concealment.

### Blinding

#### Un-blinded Site Roles

At each site, *Cognitive Remediation Coaches* are un-blinded in order to provide support for participants using their assigned programs. They will be distinct from staff administering and scoring assessments. Additionally, *Site Sub-Investigators authorized to register participants within the* TRuSST *system* will remain un-blinded and may not participate in the assessment, evaluation, or follow-up of study participants.

#### Blinded Site Roles

All site staff responsible for the administration and scoring of participant assessments will remain blinded to participant treatment. Site Principal Investigators will be required to complete a Delegation of Authority Form prior to the start of the study, indicating which activities individual site research team members will be authorized to complete. Site Principal Investigators will also remain blinded.

Depending upon the extent to which they are responsible for data collection and/or entry, *Clinical Research Coordinators* may or may not remain un-blinded to participant treatment. This will be clarified on a site-by-site basis and will be noted on the Site Principal Investigator Delegation of Authority Form.

To prevent un-blinding, the following safeguards will be instituted at each site:

1. The treatment condition and the control condition will be identified as “Treatment A” and “Treatment B”;
2. Participants will be reminded not to discuss details related to treatment with psychometricians and/or clinical evaluators during the informed consent process as well as prior to initiation and at the conclusion of each assessment visit;
3. Site personnel will be instructed to not discuss details of either treatment arm during open participant groups or forums;
4. Sites will be required to execute the protocol in a manner that minimizes the possibility of accidental un-blinding of psychometricians or clinical evaluators (e.g. unintended viewing of treatment sessions);
5. Sites will be asked to post signage in appropriate areas throughout the facility reminding staff and participants to not discuss treatment details in open locations.

At the end of the trial, psychometricians will be asked questions designed to evaluate the integrity of the blinding procedures employed throughout the trial.

### Description of Treatment Programs

#### Experimental Treatment Program (SocialVille)

**Fig. 2:**
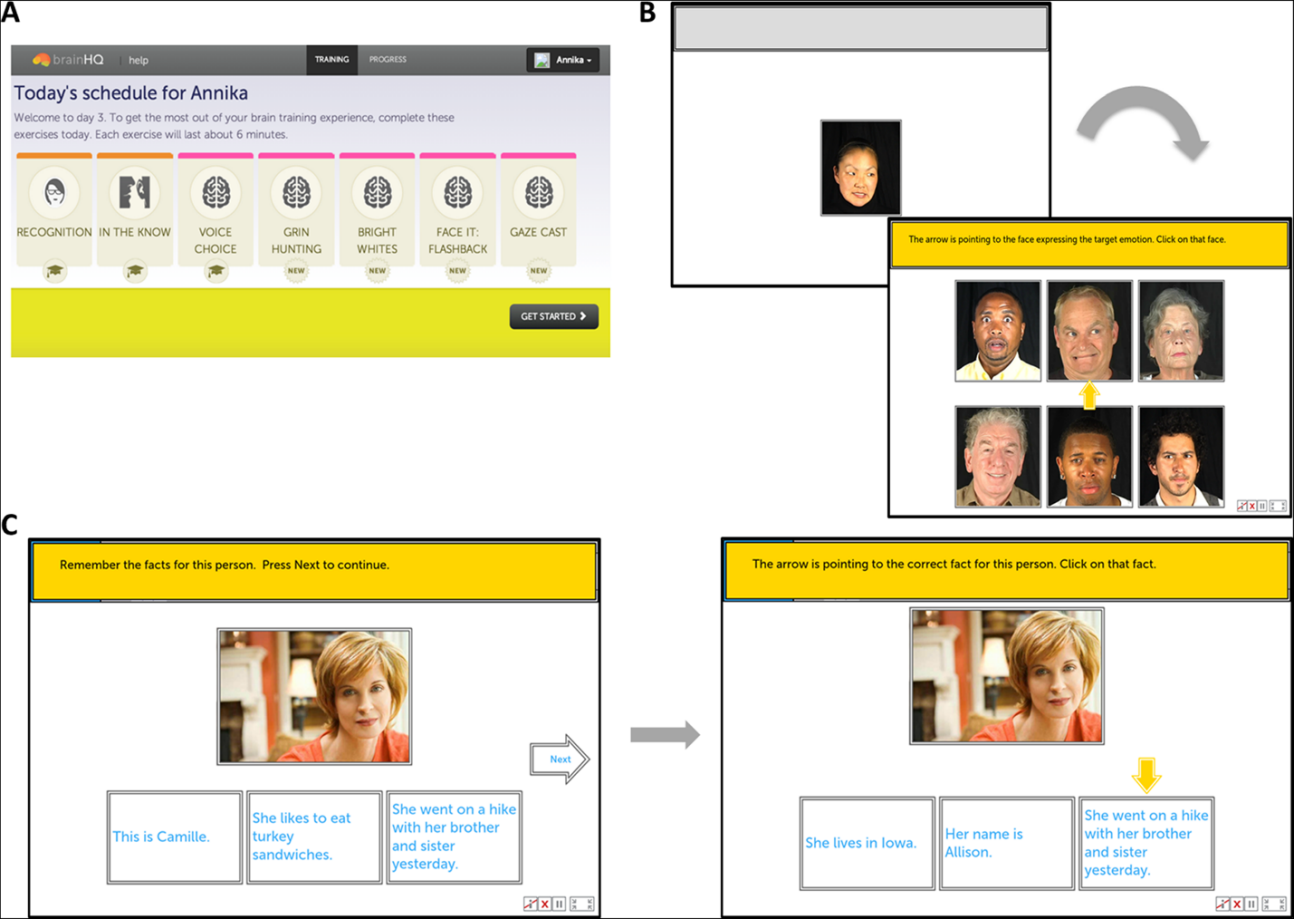
Examples of SocialVille Training Exercises. **a**. The daily schedule consists of 7 exercises of 6 minutes each, for a total of 42 minutes. **b**. Match that Feeling exercise example. In this exercise, participants are required to match the emotion depicted by the person in the target image to the emotion of a different person from a group of faces. C. Face Facts exercise example. In this exercise, participants must remember the given facts associated with a specific person

The Experimental Treatment Program (SocialVille) is a computerized social cognitive remediation program consisting of a set of specific social cognitive exercises. To use the experimental treatment program, a participant opens a standard web browser on a broadband connected computer and goes to the experimental treatment program study web site. The participant then logs into the experimental treatment program server (using a study provided login that contains no personally identifiable information). The participant completes 7 cognitive exercise blocks scheduled for the day, and performs each exercise for about 6 minutes (see Fig. 2). Participants perform tens to hundreds of trials over the course of a session, with auditory and visual feedback and rewards to indicate if the trial was performed correctly or incorrectly. After each session, the difficulty of the next session is updated (e.g., more distractors in the response array) to ensure that each participant is appropriately challenged. Summary screens including game metrics (points, levels) and exercise metrics (usage, progress) are shown to the participant at the end of each session.

All usage and progress data are encrypted then transmitted to a central server. In a research study such as this one, no personally identifiable information is stored on the server (including internet protocol addresses). On the server, the data are available for review by the un-blinded Cognitive Remediation Coach or Site Coordinator through a secure web portal. Only data from participants at a particular Site can be viewed by that Site’s staff. The Cognitive Remediation Coach in particular will use the secure web portal to regularly check on usage and progress of each active participant to customize their weekly phone/in-person discussions to provide helpful guidance and coaching.

There are multiple social cognitive exercises in SocialVille, collectively targeting the five social cognitive domains identified in the literature: affect perception, social cue perception, theory of mind (ToM), self-referential style and empathy (see Table 2 for a complete list of exercises). All exercises continuously adjust difficulty level to user performance to maintain a 70-80% correct performance rate using adaptive algorithms. The scheduling mechanism ensures that a participant progresses through the exercises in a defined order, generally moving from more simple (e.g., easy to discriminate stimulus types, less response options) exercises to more complex (e.g., greater rule complexity, greater similarity between stimuli, etc.) exercises over the course of the 8–12 weeks experience.

#### Active Control Program (commercially-available computer games)

The active control group will use conventional, progressive computer games. The 13 computer games (see Table 3 for full list) have been embedded in the training portal; this should greatly facilitate administration, and will allow maintenance of the double-blind procedure of both staff and participants, while controlling for placebo effects, time spent on computer, exposure to study staff, and non-specific effects from attended, rewarded exposure to multi-media stimulation.

This type of control is suitable for an efficacy trial, and is designed to approximate the same level of challenge as in the active training group. Moreover, it is the *only* possible control in the absence of a standardized social cognitive treatment in schizophrenia: a social skills group control would not control for number of clinic visits, interaction with study staff and other patients, and in addition it is unclear which of the potential group treatments should be chosen; A computerized social cognitive treatment is not conventionally employed, and available experimental ones critically differ from the *SocialVille* intervention in scope (targeting only a single SC domain) hence may not serve as a true control.

**Fig. 3:**
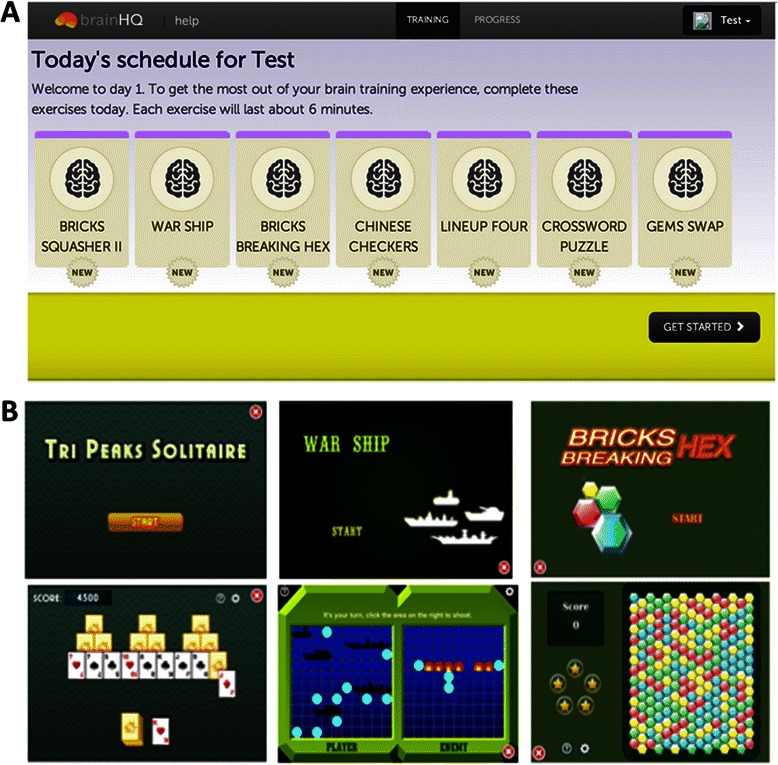
Examples of Active Control (AC) Program Training Exercises. **a**. This daily schedule for the AC program is the same as the SocialVille program, with 7 exercises presented per day for 6 minutes each. **b**. Some exercises include Tri Peaks Solitaire (3-deck solitaire), War Ship (Battleship), and Bricks Breaking Hex (remove tiles of the same color)

The active control intervention is administered in exactly the same way as the treatment intervention: it is browser-playable (from any browser) and login and password-protected. We randomly select a subset of 7 games subjects play on every session, for 6 minutes each, to equate for the time spent by the treatment group participants (see Fig. 3). The number, availability, and time spent on each game is managed by the same server which manages the treatment group exercises, hence experience is matched between the two groups. We will use games that have been shown to provide face-valid cognitive stimulation and that are rated E (for everyone) by the Entertainment Software Rating Board (ESRB).

The Cognitive Remediation Coach in particular will use the secure web portal to regularly check on usage of each active participant to customize their weekly phone/email discussions to provide helpful guidance and coaching. This ensures that the experimental treatment and active control groups are matched for social contact and encouragement from the Cognitive Remediation Coach.

### Power Calculation for Sample Size

With the aim of having 128 participants complete the study, we have statistically powered it to detect a between-groups Cohen’s d effect size of 0.50 on an outcome measure, calculated as the between-group difference in the treatment effect means (post-assessment score minus pre-assessment score) divided by the pooled standard deviation of the observed test-retest reliability. This effect size translates, for example, in an improvement of 5.8 points (on an IQ-like index score) within the treatment group versus an improvement of 1.0 point within the active control group, with both groups showing a variance of 10 points (2/3 of a standard deviation, as observed in other composite cognitive performance data) from pre-assessment to post-assessment. The results of our feasibility study [80] allowed us to derive training group effect sizes; these ranged from 0.45-1.1 on the SocialVille measures, from 0.53-0.77 for the social cognitive measures and from 0.4-0.67 for the generalization and functional measures. A recent study conducted by our study collaborator, Prof. Vinogradov [61] employed computerized social cognitive training and found effect sizes of 0.53 on the MSCEIT [94] perceiving emotions total subscale. Furthermore, a recent meta-analysis conducted on social cognitive training interventions in schizophrenia [68] found moderate to large effect sizes on facial affect recognition (Cohen’s d between 0.71-1.01) and small to moderate effect sizes on ToM (0.46). Effect size for total symptoms was found to be moderate to large (0.68). We therefore believe this is a reasonable estimate of the plausible effect size in the suggested trial, and that documentation of a 0.5 between-groups effect size in this trial would provide reasonable support for clinical benefit in this population.

### Data Analysis

For the main analysis of this study (evaluating the efficacy of SocialVille as a treatment for social cognition deficits) we will define an Intent-To-Treat (ITT) population that includes all participants who have been randomized to either group. We will compare treatment and active control groups in the ITT population to determine if any differences in baseline demographic, characterization, outcomes variables, or total program use time remain after the randomization process.

We will test the following hypotheses of the primary outcomes, in addition to exploratory analyses: (1) Experimental treatment versus active control improves social cognition, and (2) Experimental treatment versus active control improves functional capacity. In addition, we will test the following hypotheses of the secondary outcomes: (1) Experimental treatment versus active control improves symptom severity; (2) Experimental treatment versus active control improves functioning; (3) Experimental treatment versus active control improves social cognition; (4) Experimental treatment versus active control improves motivation, and (5) Experimental treatment versus active control improves quality of life.

To examine each hypothesis, we will examine the data from each outcome measure(s) associated with the Primary or Secondary outcomes using a linear mixed-effects model with group and time as fixed factors, site as a random factor, and additional factors/covariates as required if there are trends towards significant baseline differences (p < 0.1) in the treatment and active control groups. Missing data will be handled with an iterative maximum likelihood procedure to optimally estimate model parameters. The key value for significance will be the group-by-time interaction term. This modeling will be conducted with a Type I error set at 0.025 for each model.

In addition, to identify variables that predict treatment success in the SocialVille group, we will use an individual differences approach [80] and test for associations between not only outcome variables and pre-test scores (raw and composite), but also between outcome variables and improvements in aspects of the training tasks. This will help us determine if specific types of patients benefit from training more than others as well as if particular improvement patterns on training (i.e., training strategies) are predictive of successful outcomes. These analyses will be evaluated after correcting for multiple comparisons (i.e., Bonferroni).

Finally, following the main analyses, we will construct two separate composites, of low-level and high-level SC assessments, respectively, and test the effects of training on each of these composites using a linear mixed-effects model. We will further examine correlations between magnitude of change on each of the constructs and % improvement on SocialVille exercises.

## Discussion

Despite the functional significance of social cognition to everyday function, there is currently no effective and widely-adopted treatment approach for the extremely debilitating social cognition deficits seen in schizophrenia. The current TRuSST protocol shall help determine whether SocialVille is an effective treatment method for social cognition deficits in schizophrenia, promoting social and functional benefits that could potentially improve the quality of life of afflicted individuals and their family members. The fact that SocialVille is a highly-scalable intervention, deployed to any number of individuals at minimal cost and does not require additional clinical training, strengthens the notion that a successful TRuSST trial could result in the rapid utilization of SocialVille in schizophrenia treatment, creating a new and improved standard of care for this condition.

### Strengths

A major strength of the study is that it deploys simple interventions that can be administered completely remotely, with minimal remote clinical monitoring, planned for a phone call or email once/week to check in on participants and see if they encounter any difficulty completing their training. Deploying interventions remotely and saving the multiple clinical visits should potentially facilitate compliance with study requirements, and allow to mimic real-life deployment of the training program.

In addition, many previous studies and clinical trials testing social cognitive benefits in schizophrenia lacked statistical power due to relatively small sample sizes (e.g. [66, 70, 110–113]) or the lack of a control intervention (e.g. [84, 114, 115]). The TRuSST sample size, of 128 individuals, should power it to detect appropriate effect sizes. In fact, TRuSST is one of largest trials, to the best of our knowledge that were conducted in this field of social cognition in schizophrenia. The fact that it is a double-blind study further strengthens the credibility of its potential outcomes, which cannot be attributed to placebo effects or interaction with study staff.

Finally, the study employs a very large battery of outcome measures, encompassing the domains of social cognition, social functioning, quality of life and functional capacity. Specifically, the study employs some novel assessments as secondary outcome measures (e.g. VRFCAT [83]), and should thus help validate and norm these novel measures for schizophrenia, on the path of making them the new gold standard in the field.

### Weaknesses

Our protocol has some potential limitations. Due to the relatively long duration and multiple components of our study, attrition rates may pose a potential limitation, making it difficult to reach our recruitment goal. Furthermore, one of the common symptoms of schizophrenia is a lack of motivation [116, 117], which could prevent timely completions of the self-initiated training program. With a standardized protocol, frequent check-ins, and regular feedback from our research assistants, we aim to limit drop-out rate and motivate individuals to continue with training through completion.

### Trial Status

The trial is currently in the recruitment phase.

## Abbreviations

TRuSST: Treatment of Social Cognition in Schizophrenia Trial
RCT: Randomized Controlled Trial
STS: Superior temporal sulcus
mPFC: Medial Prefrontal Cortex
SCIT: Social Cognition and Interaction Training
SCST: Social Cognitive Skills Training
ETIT: Emotion and ToM Imitation Training
SCET: Social Cognition Enhancement Training
TAR: Tackling Affect Recognition
METT: Micro Expression Training Tool
VRFCAT: Virtual Reality Functional Capacity Assessment Tool
AC: Active Control
SCID-P: Structured Clinical Interview for DSM-V, Patient Edition
PANSS: Positive and Negative Syndrome Scale
WTAR: Wechsler Test of Adult Reading
C-SSRS: Columbia Suicide Severity Rating Scale
ER40: Penn Emotion Recognition Test
PROID: Prosody Identification
PFMT: Penn Facial Memory Test
MSCEIT: Mayer-Salovey-Caruso Emotional Intelligence Test
EA: Empathic Accuracy
UPSA-2: UCSD Performance-based Skills Assessment
GFS: Global Functioning Scale
SFS: Social Functioning Scale
SLOF: Specific Levels of Functioning
BIS/BAS: Behavioral Inhibition/Behavioral Activation Scale
TEPS: Temporal Experience of Pleasure Scale
QLS: Quality of Life Scale
TASIT: The Awareness of Social Interaction Test
ToM: Theory of Mind
AIHQ: Ambiguous Intentions Hostility Questionnaire
ESRB: Entertainment Software Rating Board
ITT: Intent-To-Treat
WIRB: Western International Review Board
MATRICS: Measurement and Treatment Research to Improve Cognition in Schizophreni
NIMH: National Institute of Mental Health
SC: Social Cognition
